# Do we need to change our perspective about gut biomarkers? A public data mining approach to identify differentially abundant bacteria in intestinal inflammatory diseases

**DOI:** 10.3389/fcimb.2022.918237

**Published:** 2022-11-21

**Authors:** Laura Vega, Laura Bohórquez, Juan David Ramírez, Marina Muñoz

**Affiliations:** ^1^ Centro de Investigaciones en Microbiología y Biotecnología-UR (CIMBIUR), Facultad de Ciencias Naturales, Universidad del Rosario, Bogotá, Colombia; ^2^ Molecular Microbiology Laboratory, Department of Pathology, Molecular and Cell-Based Medicine, Icahn School of Medicine at Mount Sinai, New York, NY, United States

**Keywords:** bacterial biomarkers, intestinal inflammatory diseases, differentially abundant bacteria, beneficial bacteria, pathogenic bacteria

## Abstract

**Introduction:**

The gut microbiome is involved in multiple processes that influence host physiology, and therefore, disruptions in microbiome homeostasis have been linked to diseases or secondary infections. Given the importance of the microbiome and the communities of microorganisms that compose it (microbiota), the term biomarkers were coined, which are bacteria correlated with disease states, diets, and the lifestyle of the host. However, a large field in the study of intestinal biomarkers remains unexplored because the bacterial communities associated with a given disease state have not been exactly defined yet.

**Methods:**

Here, we analyzed public data of studies focused on describing the intestinal microbiota of patients with some intestinal inflammatory diseases together with their respective controls. With these analyses, we aimed to identify differentially abundant bacteria between the subjects with the disease and their controls.

**Results:**

We found that frequently reported bacteria such as Fusobacterium, Streptococcus, and Escherichia/Shigella were differentially abundant between the groups, with a higher abundance mostly in patients with the disease in contrast with their controls. On the other hand, we also identified potentially beneficial bacteria such as Faecalibacterium and Phascolarctobacterium, with a higher abundance in control patients.

**Discussion:**

Our results of the differentially abundant bacteria contrast with what was already reported in previous studies on certain inflammatory diseases, but we highlight the importance of considering more comprehensive approaches to redefine or expand the definition of biomarkers. For instance, the intra-taxa diversity within a bacterial community must be considered, as well as environmental and genetic factors of the host, and even consider a functional validation of these biomarkers through in vivo and in vitro approaches. With the above, these key bacterial communities in the intestinal microbiota may have potential as next-generation probiotics or may be functional for the design of specific therapies in certain intestinal diseases.

## 1 Introduction

The intestinal microbiome is a modulating factor of the host’s health and proposed as an “essential organ” of the human body (Ding et al., 2019). Two concepts are fundamental to describe microbial ecology: (i) the microbiota, which refers to the communities of microorganisms belonging to different domains, and (ii) the microbiome, which considers the microbiota together with its structural elements, metabolites, and the interactions they have with the environment they inhabit (Berg et al., 2020). The main biological processes where the intestinal microbiome intervenes are the modulation of metabolism, the regulation of epithelial development, and influencing the innate immune response of the host; consequently, these processes contribute to maintaining the physiological homeostasis of the host. The disruption in physiological homeostasis and the functioning of the microbiome is usually reflected in disease states such as diabetes, inflammatory bowel disease, allergies, secondary infections by pathogens, among others (Wang et al., 2017; Feng et al., 2018).

Sequencing of the hypervariable regions of the 16s-rRNA gene through next-generation sequencing (NGS) technologies has been widely implemented for the compositional description of intestinal microbiota, in particular of the bacterial communities, which comprise a high percentage of the microbiota (10^11^- 10^12^ bacterial cells per milliliter) (Walter and Ley, 2011; Rinninella et al., 2019; Liu et al., 2021). Moreover, the compositional study of the microbiota has made it possible to identify bacterial communities related to the host’s lifestyle or state of the disease, since the abundance or even the presence of these bacteria varies according to multiple factors (host’s genetics, diet, environmental factors, etc.) (Feng et al., 2018). The study of metagenomics has even been implemented to expand the study of the microbiome because this approach allows obtaining information on the composition of the microbiota, as well as information on the functionality of the microbiome (Liu et al., 2021).

In 2011, the term enterotype was proposed to stratify the composition of the microbiota, thus establishing three well-defined and frequent bacterial communities: *Bacteroides* (Enterotype 1), *Prevotella* (Enterotype 2), and *Ruminococcus* (Enterotype 3) (Arumugam et al., 2011; Cheng and Ning, 2019; Di Pierro, 2021). However, this classification has been debated, considering that some studies did not report a structure of these three discrete clusters but a continuum of these microbial members, indicating an overlap of these clusters when spatially observed through a beta-diversity analysis (Yatsunenko et al., 2012; Koren et al., 2013; Knights et al., 2014; Costea et al., 2018). In the same way, these enterotypes present low stability in the individuals over time, thus extrapolating this concept of patterns in the microbiota to different study populations should be done cautiously (Costea et al., 2018).

With the limitations of the concept of enterotypes, the term biomarkers of the intestinal microbiota were proposed, which are understood as measurable indicators of a biological state; that is, bacterial communities that correlate with disease states, lifestyles, or diet (Gorvitovskaia et al., 2016). For example, *Faecalibacterium* is recognized as a potentially beneficial bacterium since it is a producer of butyrate (a key metabolite for maintaining intestinal homeostasis) and whose abundance is reduced in subjects with diseases such as colorectal cancer, inflammatory bowel disease, and even chronic kidney disease when compared to subjects without these conditions (Sokol et al., 2008; Jiang et al., 2016; Ferreira-Halder et al., 2017; Lopez-Siles et al., 2018). On the other hand, *Fusobacterium* has been considered as a potentially pathogenic bacteria as it has been found in high abundance in the carcinomas of patients with colorectal cancer, and therefore suggested to promote cancer progression by increasing the severity of lesions (Kostic et al., 2012; Amitay et al., 2017).

Despite that these bacterial communities are frequently reported in association with some conditions of the host, multiple factors that influence the microbiota composition should be considered in this disjunctive results. Therefore, the bacterial communities associated with a given disease state have not been exactly defined yet, which makes it essential to continue expanding the information in this study field, and thus, understand the role of some bacterial communities in different health statuses and host diseases. In addition, there are various statistical analysis methods (DESeq2, LEfSe, ALDEx2, ANCOM, etc.) that are implemented by studies to identify potential biomarkers of the intestinal microbiota, which leads to variable results when it comes to analyzing communities of importance in some host states (Nearing et al., 2022). Thus, this study aims to analyze those taxa that may present a differential abundance between subjects with an inflammatory bowel disease compared to subjects without the disease. The results of this study would support previous findings that have described the potential beneficial or pathogenic role of certain bacteria associated with disease states. Likewise, these differentially abundant bacteria can be proposed as next-generation probiotics (NPGs), considering that those pathogenic communities could be useful for the design of disease diagnostic tests, while beneficial communities could be implemented to promote health states in the host.

## 2 Materials and methods

### 2.1 Public data retrieval and construction of the database

The search of scientific articles that complied with the inclusion criteria was carried out in PubMed implementing the following search algorithm: “gut microbiota”[Title/Abstract] AND “disease of interest”[Title/Abstract]) NOT (review). This query was carried out between February of 2021 and September of 2022. Only studies whose sequencing data were publicly available were considered. From the studies retrieved in the query, we reviewed those that seemed to comply with the established criteria and were subsequently organized in a database that contained 10 categories: a) Author(s), b) Title of the study, c) Year of publication, d) Disease of interest, e) Public repository, f) Study accession number in the public repository, g) Sample type, h) Sequencing platform, i) 16s-rRNA hypervariable region, and j) Set of primers implemented in the study. Some categories will be detailed below:

#### 2.1.1 Public repository

This refers to the repository in which the study data is available and could be downloaded. In particular, the most frequent repositories were the European Nucleotide Archive and the Sequence Read Archive of the NCBI. Each study had a unique accession number that allowed us to locate it in the repository.

#### 2.1.2 Sample type

This refers to the sample collected in the study, and that allowed the description of gut bacterial communities. Here we considered either biopsies or stool samples from the cases and controls.

#### 2.1.3 Sequencing platform

Corresponds to the next generation sequencing platform employed in the study. According to our inclusion criteria, only Illumina or Roche 454 was considered since Illumina is one of the most used platforms for microbiota analysis, because it generates thousands of reads per sample, the error rate is lower than that third-generation sequencing platforms and the bioinformatic analysis are relatively standardized (Liu et al., 2021). We also considered Roche 454 to examine a larger number of studies, and consequently include more data to the final dataset.

#### 2.1.4 Hypervariable region and set of primers

Ribosomal gene 16s-rRNA has nine hypervariable regions (V1-V9) flanked by highly conserved regions (Barb et al., 2016). The studies that describe the bacterial communities of the microbiota can target any region to perform the taxonomic assignation of the reads resulting from a sequencing process, although the most targeted region, and that retrieves high quality results is the V4 region. (Kim et al., 2011; Gursoy and Can, 2019). In each study, the authors implemented a pair of primers for sequencing the selected hypervariable region of the 16s-rRNA to describe the bacterial communities. Here, we did not restrict the query to a particular hypervariable region, as it would restrict the amount of data.

### 2.2 Inclusion and exclusion criteria

For this study, we considered studies that enrolled adult subjects with the following intestinal inflammatory diseases: Colorectal cancer (CRC), Crohn’s disease (CD), Ulcerative colitis (UC), Irritable bowel syndrome (IBS), *Clostridioides difficile* infection (CDI), and their healthy control subjects (HC, subjects without the disease). Thus, the systematic review of the literature was focused on studies that aimed to describe the gut prokaryotic communities of the cases and control groups using next-generation sequencing (Illumina platform or Roche 454). Specifically, we included those articles that employed amplicon-based sequencing of different hypervariable regions of the 16s-rRNA. In addition, we considered studies that collected biopsies or fecal samples from the subjects. Conversely, those articles that considered patients with more than two diseases at the time were excluded from the analysis. In the same way, we excluded studies that implemented treatments upon the subjects with the disease. However, we considered longitudinal studies retrieving only the sequences of the first timepoint to have a robust database.

### 2.3 Bioinformatic and statistical analyses

The publicly available sequences from each study were downloaded for subsequent quality control and taxonomic allocation. Initially, we performed quality control of the sequences using FastQC and MultiQC (Andrews, 2010; Ewels et al., 2016). Later, the barcodes were removed from the sequences using QIIME2 if required (Bolyen et al., 2019). The taxonomic assignation of these sequences was performed with the R package DADA2, implementing the recommended pipeline for microbiome analysis (https://benjjneb.github.io/dada2/tutorial.html) (Callahan et al., 2016). Initially, individual reads were filtered considering a Phred score between 20 and 30 and then forward and reverse reads were merged. Subsequently, the algorithm inferred and constructed a table of the Amplicon Sequence Variant (ASV), which was used for the elimination of chimeras in the sequences. Finally, these ASVs were subjected to the process of taxonomic allocation implementing the reference database SILVA v138.1 (McLaren and Callahan, 2021). Sequencing depth normalization of the considered studies was not performed, as it would involve a loss in the information provided by each study (Gloor et al., 2017; Willis, 2019).

For the descriptive analyses of the microbiota composition, we generated a global dataset for each disease, consolidating the results of taxonomic assignment of the studies included in each disease. The phyloseq package was implemented for preprocessing of the data, such as normalizing read counts and merging ASVs at a determined taxonomic rank with the *tax_glom* function (McMurdie and Holmes, 2013). The Abundance-based Coverage Estimator (ACE) was calculated for determining the richness estimation of the bacterial communities, and Shannon-Weaver and Simpson indexes were calculated to estimate the diversity of the communities within the study groups. Statistical differences of richness and diversity between the two study groups were assessed with a Mann-Whitney test or a T-test (p-value <0.05), according to normality tests. For beta diversity analysis we performed a principal coordinate analysis, with Bray-Curtis calculated distances and implementing a permutational analysis of variance using distance matrices (adonis) to evaluate statistical differences of the clustering. The phyloseq package was implemented for the mentioned analyses and to describe the taxonomic composition at the phylum level in the two study groups of each disease. The description of the phyla was performed for each disease, considering all the studies that comprise it. Additionally, all the phyla with less than 100 reads were grouped under the “Others” category. Statistical tests for the most dominant phyla between the two study groups were assessed with a Mann-Whitney test, considering a p-value< 0.05. A particular case was presented for the studies of CDI, where only one of the selected studies could be analyzed descriptively (Duan et al., 2020); thus, this disease was discarded for subsequent analyses.

Afterward, we performed the framework of analysis of composition of microbiomes (ANCOM) to identify the genera with a differential abundance between the two groups (cases and controls) considered in each study (Mandal et al., 2015; Nearing et al., 2022). For the ANCOM we considered a threshold of 0.7 and a p-value <0.05. Thus, those bacterial genera with a statistically significant difference in their abundance between the controls and cases were selected. As a result, if any differentially abundant bacterial genus was shared in two or more studies, it was considered relevant to intestinal inflammatory diseases. To facilitate the visualization of the ANCOM results, we calculated the relative abundance of each genus, considering the reads of the genera with a significant difference in each study group (cases and controls) as the total. Statistical analyzes and generation of the corresponding figures were all performed using the R v.4.1.0, along with the packages reshape2, tidyverse, ggplot2 and hrbrthemes (R Core Team, Vienna, Austria). The **Figure S1** offers a brief description of data retrieval, bioinformatic, and statistical analyses mentioned here (**Figure S1**).

## 3 Results

### 3.1 Dataset description

The PubMed query yielded a total of 563 articles for colorectal cancer, 361 for Crohn’s disease, 582 for ulcerative colitis, 286 for irritable bowel syndrome, and 177 for *Clostridioides difficile* infection, obtaining a total of 1,969 articles. Due to the following reasons, some articles were discarded: the study did not meet the inclusion criteria, the metadata provided by the study did not enable the differentiation of case and control samples, the accession number corresponding to the sequencing data of the study was not found in the repositories, and the quality of the sequences was too low for downstream analysis. After inclusion criteria evaluation, quality verification, and taxonomic assignment with DADA2, the total of analyzed studies were: five for CRC (Lu et al., 2016; Clos-Garcia et al., 2020; Uchino et al., 2021; Du et al., 2022; Konishi et al., 2022), eight for CD (Forbes et al., 2018; Zhou et al., 2018a; Braun et al., 2019; Lloyd-Price et al., 2019; Weng et al., 2019; Alam et al., 2020; Bourgonje et al., 2022; Pisani et al., 2022), nine for UC (Bajer et al., 2017; Halfvarson et al., 2017; Forbes et al., 2018; Zhou et al., 2018a; Lloyd-Price et al., 2019; Weng et al., 2019; Alam et al., 2020; Quraishi et al., 2020; Bourgonje et al., 2022; Pisani et al., 2022), four for IBS (Zhuang et al., 2018; Zhu et al., 2019; Wang et al., 2020; Rogers et al., 2021), and one for CDI Duan et al., (2020). Additional information on the mentioned studies is described in **Supplementary Data 1**.

### 3.2 Subjects with colorectal cancer display higher bacterial richness than control subjects

**Figure 1:**
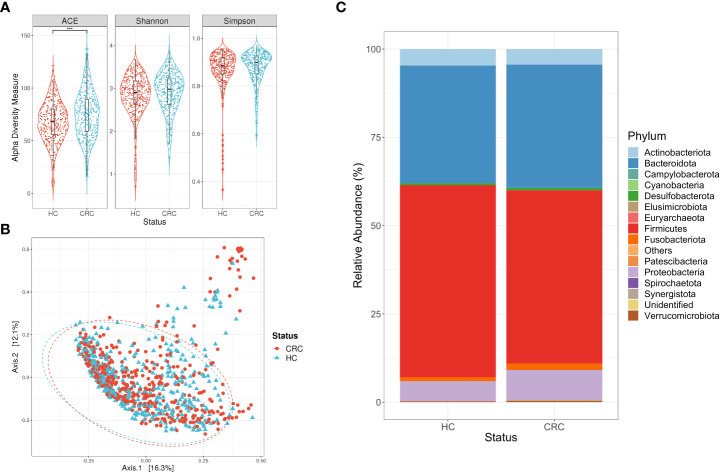
Descriptive analysis for the consolidation of studies focused on colorectal cancer (CRC). **(A)** Alpha diversity measures calculated for the ASVs of subjects with CRC and their respective controls (HC). Statistical differences were evaluated with a T test (***p-value <0.001). **(B)** Principal Coordinate Analysis (PCoA) for the ASVs of subjects with CRC and their respective controls (HC). Statistical differences of beta diversity were evaluated with the adonis test (r^2 =^ 0.037, p-value <0.001). **(C)** Relative abundance of the phyla for each of the study groups, being Firmicutes, Bacteroidota, and Proteobacteria the predominant phyla.

The ACE richness estimator had higher values in the group of subjects with colorectal cancer compared to control subjects (p-value <0.001, [T test]). Alternatively, we observed homogeneous results for the diversity indexes between the two study groups (p-value >0.05, [Mann-Whitney]) (**Figure 1A**). Despite that the adonis test showed a significant result for the PCoA (r^2 =^ 0.01055, p-value <0.001), the clusters of the disease and control groups spatially overlapped with each other. In addition, the most abundant phyla in both groups were Firmicutes, Bacteroidota, and Proteobacteria, respectively. It should be noted that the phylum Proteobacteria exhibited a lower relative abundance in the group of control subjects than in the group of subjects with the disease (p-value <0.001, [Mann-Whitney]). In contrast, the phylum Firmicutes was found in a higher abundance within the control subjects compared to the colorectal cancer subjects (p-value <0.001) [Mann-Whitney]. Finally, we did not observe significant differences in the abundance of Bacteroidota phylum between the two study groups (p-value >0.05, [Mann-Whitney]) (**Figure 1C**).

### 3.3 Diminished bacterial diversity in a Crohn’s disease scenario

**Figure 2:**
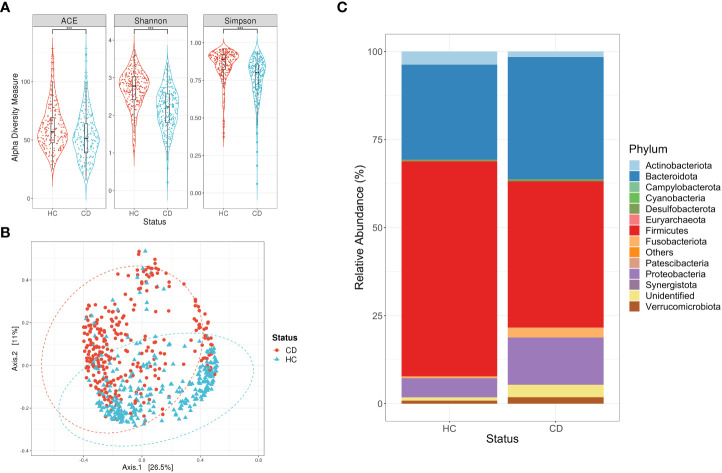
Descriptive analysis for the consolidation of studies focused on Crohn’s disease (CD). **(A)** Alpha diversity measures calculated for the ASVs of subjects with CD and their respective controls (HC). Statistical differences were evaluated with a Mann-Whitney test and a T-test (***p-vlaue <0.001). **(B)** Principal Coordinate Analysis (PCoA) for the ASVs of subjects with CD and their respective controls (HC). Statistical differences of beta diversity were evaluated with the adonis test (r^2 =^ 0.025, p-value <0.001). **(C)** Relative abundance of the phyla for each of the study groups, where Firmicutes is significantly reduced in CD subjects.

In general, subjects with Crohn’s disease exhibited a lower richness of bacterial ASVs, than their corresponding controls (p-value <0.0001, [Mann-Whitney]). In the same way, the Shannon and Simpson indexes displayed a low bacterial diversity in the subjects with Crohn’s disease compared to the control group (p-value <0.0001, [Mann-Whitney]) (**Figure 2A**). The principal coordinate analysis (PCoA) displayed a differential clustering between the samples of the subjects with colorectal cancer and their corresponding controls (adonis, r^2 =^ 0.05543, p-value <0.001), which suggests the presence of some bacterial communities that differentiate one group from the other (**Figure 2B**). The predominant phyla in both groups were Firmicutes, Bacteroidota, and Proteobacteria, and all displayed significant differences in their abundance between the study groups. For instance, Firmicutes had a lower abundance in the subjects with Crohn’s disease in contrast to the controls (p-value <0.001, [Mann-Whitney]). On the other hand, we noted a higher abundance of Bacteroidota and Proteobacteria within the group of subjects with the disease compared to their controls (p-value <0.001, [Mann-Whitney]) (**Figure 2C**).

### 3.4 Ulcerative colitis and control subjects display a similar bacterial richness and abundance of Bacteroidota

**Figure 3:**
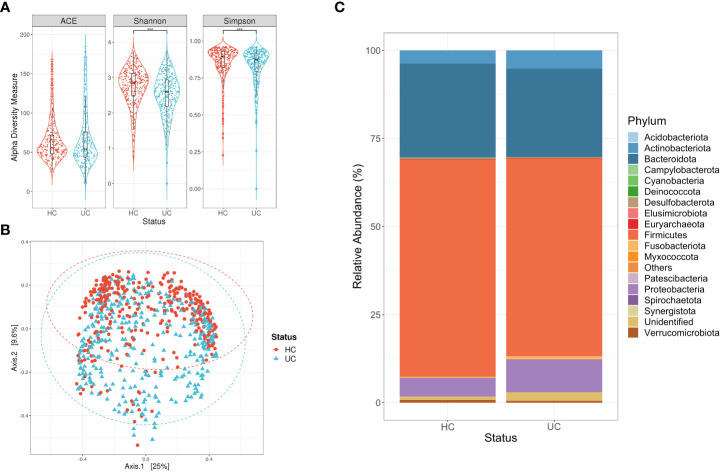
Descriptive analysis for the consolidation of studies focused on ulcerative colitis (UC). **(A)** Alpha diversity measures calculated for the ASVs of subjects with UC and their respective controls (HC). Statistical differences were evaluated with a Mann-Whitney test (***p-vlaue <0.001). **(B)** Principal Coordinate Analysis (PCoA) for the ASVs of subjects with UC and their respective controls (HC). Statistical differences of beta diversity were evaluated with the adonis test (r^2 =^ 0.012, p-value <0.01). **(C)** Relative abundance of the phyla for each of the study groups, where Proteobacteria displays a higher abundance in UC subjects.

The alpha diversity measures showed a similar bacterial richness and a significant difference in the Shannon and Simpson indexes between the groups, where a low diversity was observed in the subjects with ulcerative colitis (p-value <0.05, [Mann-Whitney]) (**Figure 3A**). Alternatively, the PCoA did not display a differential clustering of the two groups, despite the adonis test yielding a significant result (adonis, r^2 =^ 0.01644, p-value <0.01) (**Figure 3B**). In this case, we also found that the predominant phyla were Firmicutes, Bacteroidota, and Proteobacteria, where **Figure 3C** illustrates a similarity in the abundance of Bacteroidota between the two study groups (p-value> 0.01, [Mann-Whitney]). However, the statistical tests showed a significantly higher abundance of Firmicutes in the control subjects compared to those with ulcerative colitis (p-value< 0.05, [Mann-Whitney]). Finally, we noted a significantly higher abundance of Proteobacteria in the subjects with ulcerative colitis compared to the control subjects (p-value< 0.001, [Mann-Whitney]).

### 3.5 Irritable bowel subjects had a lower abundance of Actinobacteria compared to their corresponding controls

**Figure 4:**
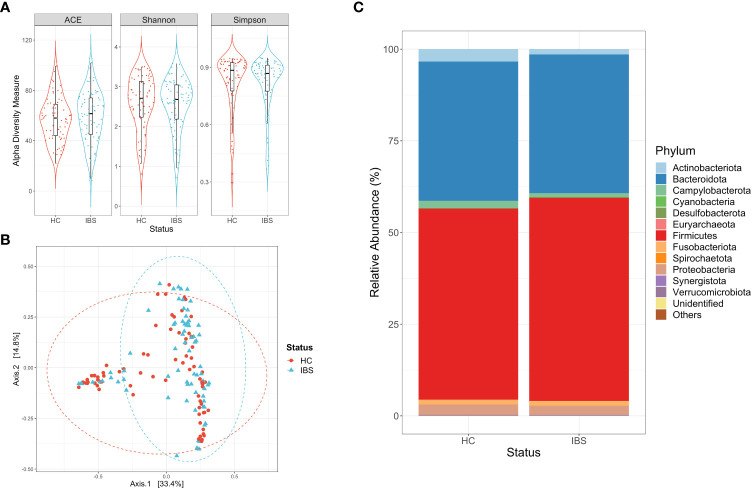
Descriptive analysis for the consolidation of studies focused on irritable colon syndrome (IBS). **(A)** Alpha diversity measures calculated for the ASVs of subjects with IBS and their respective controls (HC). Statistical differences were evaluated with a Mann-Whitney test (p-value >0.05). **(B)** Principal Coordinate Analysis (PCoA) for the ASVs of the group of subjects with the disease and their respective controls. Statistical differences of beta diversity were evaluated with the adonis test (r^2 =^ 0.023, p-value >0.01). **(C)** Relative abundance of the phyla for each of the study groups, being Firmicutes, Bacteroidota, and Actinobacteriota the predominant phyla.

The alpha diversity measures showed similar values of bacterial richness and diversity between the IBS subjects and their controls (**Figure 4A**). Regarding the beta diversity analysis, we noted a clustering of the study groups, and the adonis test yielded a significantly different distribution of the clusters (adonis, r^2 =^ 0.023, p-value >0.01) (**Figure 4B**). In the case of this disease, the predominant phyla were Firmicutes and Bacteroidota, and the abundance of these phyla was similar between the two study groups (p-value >0.05, [Mann-Whitney]) (**Figure 4C**). On the contrary, a low abundance of the phylum Actinobacteriota was observed in the IBS subjects in contrast with the controls (p-value< 0.01, [Mann-Whitney]) (**Figure 4C**).

The previous results show that the phyla Firmicutes and Proteobacteria can be found differentially in the study groups, which would suggest a potentially relevant role of these phyla in intestinal diseases. However, within some phyla, such as Firmicutes, there is a wide diversity of genera with functional differences in the microbiota (Gorvitovskaia et al., 2016). Thus, the analyses described below focused on identifying potential members with importance in the intestinal microbiota, considering a resolution at the genus level, which allows exploring the differences found at the phylum level with more specificity.

### 3.6 Differentially abundant genera in the intestinal inflammatory diseases

The ANCOM workflow was performed for the genera identified in the study groups of each disease to increase the resolution of the analysis about the candidates that may be relevant in intestinal diseases. **Table 1** includes the studies that presented a significant difference in the abundance of some bacterial genera between the two groups (cases and controls), which means that the ANCOM prompted a p-value <0.05 and a threshold of 0.7. Some of the studies analyzed were not included in **Table 1** because no significant differences were found in the abundance of the genera when performing the ANCOM (p>0.05).

**Table 1:** Genera with differential abundance between the study groups (cases and controls) in each of the analyzed studies.

Disease	Study	Genus	Abundance within disease group (%)	Abundance within control group (%)	W
CRC	Clos-Garcia et al. (2020)	*Rikenellaceae* RC9 gut group	35.65	9.92	151
		*Anaerostipes*	6.3	20.37	177
		Blautia*	29.14	63.6	140
		*Prevotella*	22.71	5.5	139
		*Fusobacterium* ^†^	6.2	0.61	148
	Lu et al. (2016)	*Acinetobacter*	0.68	0	41
		*Bacillus*	0.11	15.76	52
		*Brochothrix*	3.29	0	52
		*Enhydrobacter*	4.05	0	51
		*Enterococcus*	0	65.82	52
		*Flavobacterium*	0.44	0	38
		*Halomonas*	1.14	0	39
		*Janthinobacterium*	0.67	0	46
		*Lactococcus*	58.63	7.27	43
		*Leuconostoc*	1.95	0	51
		*Lysinibacillus*	0	2.64	48
		*Myroides*	1.6	0	48
		*Paenisporosarcina*	0	0.09	40
		*Pseudomonas* ^†^	22.32	0	52
		*Psychrobacter*	3.72	0	51
		*Solibacillus*	0	6.13	52
		*Sporosarcina*	0	1.99	48
		*Streptococcus* ^†^	1.41	0.31	49
	Du et al. (2022)	*Eisenbergiella*	98.98	1.01	93
		*Peptostreptococcus*	96.74	3.26	101
	Konishi et al. (2022)	*Fusobacterium*	59.68	40.32	157
		*Agathobacter*	39.83	60.17	138
		*Fusicatenibacter*	43.28	56.72	130
		*Parasutterella*	33.89	66.11	140
		*Parvimonas*	99.45	0.55	160
		*Roseburia*	41.08	58.91	116
		*Monoglobus*	35.81	64.19	139
		*[Eubacterium] ventriosum group*	39.57	60.43	132
		*Butyricicoccus*	40.75	59.25	145
		*Lachnospira*	31.23	68.77	158
		*Porphyromonas*	99.57	0.42	122
		*Gemella*	96.38	3.62	157
		*Peptostreptococcus*	99.89	0.11	160
		*Solobacterium*	97.39	2.61	132
		*Eisenbergiella*	61.67	38.33	121
	Uchino et al. (2021)	*Fusobacterium*	64.27	35.73	138
		*Bifidobacterium*	32.08	67.92	146
		*Parvimonas*	99.92	0.08	146
		*Peptostreptococcus*	99.91	0.09	138
CD	Alam et al. (2020)	Faecalibacterium*	17.53	82.47	92
	Braun et al. (2019)	*Escherichia-Shigella* ^†^	97.55	43.84	127
		*Ruminococcus*	2.45	56.16	104
	Forbes et al. (2018)	*Streptococcus*	42.73	26.57	103
		*Faecalibacterium*	2.62	38.73	119
		*[Ruminococcus] gnavus* group^†^	10.43	2.5	135
		*Veillonella*	29.66	0.09	98
		*Erysipelatoclostridium*	1.69	1.91	112
		*[Clostridium] innocuum group* ^†^	3.58	0.07	129
		*Christensenellaceae* R-7 group	0.67	12.6	119
		*Eggerthella* ^†^	1.77	0.99	106
		*Marvinbryantia*	0.48	8.09	112
		*Lachnoclostridium*	4.25	1.25	129
		Sellimonas*	1.13	0.3	113
		*UCG-002*	0.71	6.82	103
		*Faecalitalea*	0.28	0.06	107
	Weng et al. (2019)	*Fusobacterium*	91.59	31.19	94
		Roseburia*	1.53	34.4	104
		*Lachnospira*	2.22	11.5	86
		*Subdoligranulum*	2.69	12.87	84
		*Ruminococcus*	1.97	10.04	81
	Lloyd-Price et al. (2019)	*Dialister*	99.90	0.0098	141
		*UCG-002*	8.60	91.39	122
		*Lachnospiraceae* UCG-010	11.89	88.11	117
		*UCG-005*	28.10	71.89	116
		*Barnesiella*	3.09	96.90	107
		*Christensenellaceae* R-7 group	49.47	50.53	119
	Pisani et al. (2022)	*Dialister*	34.66	65.33	92
		*Flavonifractor*	49.26	50.74	98
		*Oscillibacter*	43.28	56.71	98
	Bourgonje et al. (2022)	*Subdoligranulum*	10.60	89.39	90
		*Escherichia-Shigella*	78.54	21.46	74
		*UCG-002*	1.76	98.24	91
		*Romboutsia*	2.66	97.34	87
		*[Ruminococcus] gnavus* group	91.98	8.01	94
		*Bacteroides*	3.98	96.02	79
		*UCG-005*	3.77	96.22	76
		*Erysipelatoclostridium*	98.18	1.82	97
		*Sellimonas*	100.00	0.00	96
		*Tyzzerella*	67.44	32.56	73
		*Gemella*	100.00	0.00	80
		*Christensenellaceae* R-7 group	4.21	95.79	89
		*Lactobacillus*	33.01	66.90	72
		*Streptococcus*	72.96	27.03	97
		*[Eubacterium] brachy* group	75.87	24.12	74
		*UBA1819*	46.57	53.43	70
		*Lachnoclostridium*	68.05	31.94	93
		*Veillonella*	100.00	0.00	85
		*Faecalibacterium*	11.67	88.33	83
UC	Bajer et al. (2017)	Phascolarctobacterium*	34.3	44.62	94
		*[Ruminococcus] torques*group	65.7	55.38	101
	Forbes et al. (2018)	*[Clostridium] innocuum*group	97.16	2.88	122
	Halfvarson et al. (2017)	*Prevotella_9*	99.77	99.7	152
		*Lachnospiraceae* AC2044 group	0.23	0.3	147
	Quraishi et al. (2020)	*Salmonella*	0	100	167
	Weng et al. (2019)	*Desulfovibrio*	3.26	96.74	108
	Lloyd-Price et al. (2019)	*Alistipes*	29.19	60.81	118
		*UCG-002*	7.49	92.50	128
		*Bilophila*	19.72	80.28	125
		*UCG-005*	12.18	87.81	117
		*Christensenellaceae*R-7 group	11.38	88.62	125
		*Ruminococcus*	20.95	79.05	132
	Pisani et al. (2022)	*Acidaminococcus*	83.44	16.56	119
		*Phascolarctobacterium*	32.24	67.76	105
		*UCG-002*	29.82	70.18	109
		*Akkermansia*	8.59	91.40	113
		*Flavonifractor*	70.02	29.98	103
		*UCG-005*	33.46	66.53	85
		*Christensenellaceae*R-7 group	25.48	74.52	95
	Bourgonje et al. (2022)	*Subdoligranulum*	35.28	64.72	74
		*Romboutsia*	31.58	68.41	68
		*UCG-002*	12.70	87.29	78
		*Fusicatenibacter*	30.73	69.26	77
		*[Ruminococcus] gnavus*group	97.62	2.38	79
		*Christensenellaceae*R-7 group	17.89	82.11	83
		*Bacteroides*	24.04	75.96	75
		*Erysipelatoclostridium*	98.48	1.51	81
		*Alistipes*	16.92	83.08	69
		*Lachnospiraceae* NK4A136 group	17.65	82.35	68
		*UCG-005*	18.42	81.58	71
		*Lachnoclostridium*	86.41	13.59	73
		*NK4A214* group	12.39	87.60	72
		*Lactobacillus*	78.21	21.79	69
		*Veillonella*	100.00	0.00	62
		*Streptococcus*	86.54	13.46	88
		*Faecalibacterium*	30.50	69.50	85
IBS	Zhu et al. (2019)	*Intestinibacter*	17.63	1.51	133
		*Clostridium sensu stricto 1*	28.12	3.16	126
		*[Ruminococcus] gnavus* group	19.94	2.78	114
		*Erysipelotrichaceae* UCG-003	7.4	2.24	101
		*Prevotella_9*	15.38	51.58	123
		*Terrisporobacter*	5.74	0.06	125
		*Parasutterella*	0	8.99	115
		*Streptococcus*	0	7.77	135
		*Escherichia-Shigella*	0	5.96	138
		*[Eubacterium] eligens* group	2.01	0.33	108
		*Lachnospiraceae* ND3007 group	1.17	0.58	114
		*Shuttleworthia*	0.83	0	111
		*CAG-352*	1.2	5.56	120
		*Dialister*	0	5.04	120
		*Phascolarctobacterium*	0	2.82	132
		*Lachnospiraceae* NK4A136 group	0.58	0.39	102
		*Turicibacter*	0.01	0.73	99
		*Odoribacter*	0	0.48	100

Here, all the studies are shown by disease state. The relative abundance that each genus presented within the group of interest is displayed, together with the W statistic produced by the ANCOM.

*Examples of bacterial genera considered as beneficial microorganisms of the gut microbiota.

^†^Examples of bacterial genera considered as potential pathogens of the gut microbiota.

**Figure 5:**
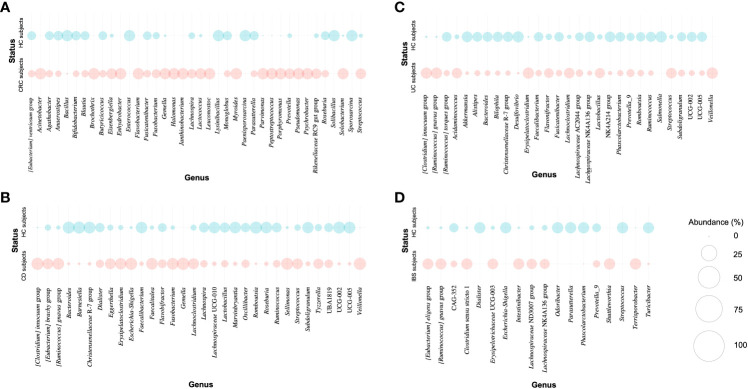
Bubble plot for the relative abundance of the genera with a significantly differential abundance for each study. **(A)** Differentially abundant genera in CRC subjects and their corresponding controls (HC). **(B)** Differentially abundant genera in CD subjects and their corresponding controls (HC). **(C)** Differentially abundant genera in UC subjects and their corresponding controls (HC). **(D)** Differentially abundant genera in IBS subjects and their corresponding controls (HC). The relative abundance of each genus was calculated by totalizing the number of reads that the genus presented in each of the studies and considering the reads in this group of genera with differential abundance as the total.

In general, there was a higher abundance of some bacterial genera considered pathogenic in subjects who presented any of the diseases compared to healthy controls (**Table 1**). In the case of subjects with colorectal cancer, there was an enrichment of *Fusobacterium* and *Streptococcus*, whereas some bacterial considered beneficial, such as *Blautia*, were decreased (**Figure 5A**). Also, we noted that in three out of five colorectal cancer studies, the genus *Peptostreptococcus* was significantly higher in colorectal cancer subjects compared with the control subjects. A similar scenario was observed in Crohn’s disease studies, where some bacteria of relevance (i.e., *Faecalibacterium*, *Christensenellaceae R-7* group, and *Roseburia*) were diminished in the Crohn’s disease subjects compared to the control subjects. In contrast, the subjects with Crohn’s disease had enrichment of bacteria such as *Fusobacterium*, *[Clostridium] innocuum group*, *[Ruminococcus] gnavus group*, *Eggerthella*, *Escherichia-Shigella* and *Streptococcus* (**Figure 5B**).

Correspondingly, we observed a lower abundance of *Faecalibacterium*, *Christensenellaceae R-7* group, and *Akkermansia* in subjects with ulcerative colitis compared to the control subjects. Additionally, the data showed a statistically significant increase in *[Ruminococcus] gnavus group*, *[Clostridium] innocuum group*, and *Streptococcus* in the ulcerative colitis subjects compared to their controls (**Figure 5C**). Finally, the genera *[Eubacterium] eligens group*, *[Ruminococcus] gnavus group*, and *Lachnospiraceae NK4A136* group exhibited increased abundance within the subjects with irritable bowel syndrome. We observed a decrease in *Escherichia-Shigella* and *Streptococcus* in subjects with irritable bowel syndrome compared to their respective controls, which is interesting given that these two genera were increased within the groups of colorectal cancer and Crohn’s disease subjects (**Figure 5D**).

The previous results based on the ANCOM allowed us to identify the bacterial genera that frequently displayed a significant differential abundance between the two study groups of each disease. The identified genera were as follows: *Streptococcus*, *[Clostridium] innocuum group*, *[Ruminococcus] gnavus group*, *Fusobacterium*, *Prevotella_9*, *Escherichia-Shigella*, *Phascolarctobacterium*, *Faecalibacterium*, *Roseburia*, *Christensenellaceae R-7* group, *Lactobacillus*, *Flavonifractor*, *Parasutterella*, *Lachnospira*, *Gemella*, *Ruminococcus*, *Veillonella*, *Erysipelatoclostridium*, *Lachnoclostridium*, *UCG-002*, *UCG-005*, *Subdoligranulum*, *Dialister*, *Romboutsia*, and *Bacteroides*. The mentioned bacterial genera could play crucial roles in the intestinal microbiota in scenarios of intestinal inflammatory diseases. Furthermore, we compared the results from the original study with those obtained herein considering the differentially abundant bacteria in the two study groups. For instance, colorectal cancer was the disease with the highest concordance between the genera identified as differentially abundant, both in the original study and this study. In contrast, ulcerative colitis was the disease with the least concordance between the results from the original study and those found herein (**Supplementary Data 2**).

## 4 Discussion

The term enterotype was initially implemented to describe three frequent and apparently well-defined bacterial populations of the intestinal microbiota. However, it has been suggested that this concept would not be appropriate to predict a disease state, considering that this clustering may mask key taxa in these types of states (Knights et al., 2014; Cheng and Ning, 2019). Therefore, Gorvitovskaia et al., 2016 proposed the term biomarkers since these function as measurable indicators of a biological state and can be considered as bacterial communities related to a disease state or lifestyle of the host (Gorvitovskaia et al., 2016). However, the identification and characterization of biomarkers of the intestinal microbiota that may be associated with particular disease scenarios is an extensive field that is still under study. For this reason, the present study aimed to propose some differentially abundant bacteria with relevance in four states of intestinal disease: colorectal cancer, Crohn’s disease, ulcerative colitis, and irritable bowel syndrome.

Alpha and beta diversity estimation of the microbiota data is a first approximation to differentiate its composition, in this case, between the subjects with disease and their controls. Initially, the subjects with Crohn’s disease had low richness and diversity of bacterial ASVs compared to their control group (**Figure 2A**), which is consistent with what has been reported in other studies (Sankarasubramanian et al., 2020). Conversely, the alpha diversity measures calculated for subjects with colorectal cancer (**Figure 1A**), ulcerative colitis (**Figure 3A**), and irritable bowel syndrome (**Figure 4A**) displayed similar or higher bacterial diversity in contrast to their respective control group. A higher diversity of microorganisms has been reported in subjects with colorectal cancer due to the exacerbated increase of pathogenic bacteria instead of beneficial bacteria in the microbiota. This hypothesis can also be adjusted to the ulcerative colitis subjects, where the same scenario was observed (Feng et al., 2015). In general, the PCoA plots did not show a defined clustering between the two study groups, despite prompting a significant result in the adonis test (**Figure 1B**–**Figure 4B**). The previous statement can be explained based on the test’s principle, where a significant difference is shown if there is a difference in the dispersion of the centroids (Anderson and Walsh, 2013). Finally, our results on the description of the dominant phyla on the study groups (cases and controls) are consistent with what was proposed in previous studies, where it is described that the phyla Firmicutes and Bacteroidota are the most abundant and frequent in the intestinal microbiota (Dave et al., 2012; Zou et al., 2019).

The results obtained by the ANCOM allowed the identification of 25 bacterial taxa that often had a differential abundance between the two study groups considered in inflammatory bowel diseases; therefore, they could be relevant in the status of these diseases. A similar study developed by Mancabelli et al., 2017 identified some potential universal biomarkers corresponding to genera associated with a healthy state of the subjects (i.e., *Barnesiella*, *Alistipes*, *Ruminococcaceae UCG-005*, among others), as well as genera that were increased in subjects with any intestinal inflammatory disease (i.e., *Streptococcus*) (Mancabelli et al., 2017). This contrasts with the results of the present study, where the *Streptococcus* genus was also found to be increased in subjects with colorectal cancer (**Figure 5A**), Crohn’s disease (**Figure 5B**), and ulcerative colitis (**Figure 5C**). In particular, *Streptococcus gallolyticus* subsp. *gallolyticus* has been found in patients with colorectal cancer and inflammatory bowel disease, and it has been suggested that these subspecies promote the development of colorectal cancer by promoting cell proliferation *via* the β-catenin pathway (Kumar et al., 2018; Sheikh et al., 2020). The previous validates our findings of *Streptococcus* as a potentially relevant genus in diseases such as colorectal cancer or Crohn’s disease so that future studies can focus on the identification and characterization of these subspecies in subjects with these diseases. Moreover, metabolic, and transcriptomic profiling of the host could be carried out to understand how the presence of this bacteria affects the host metabolic pathways and cellular processes that may exacerbate or promote this state of disease.

Further, our results highlight what was proposed in previous studies, where a decrease in beneficial bacteria has been observed, whereas there is an enrichment of potentially pathogenic bacteria in certain disease states (**Table 1**) (Karlsson et al., 2012; Feng et al., 2017; Wang et al., 2017; Lopez-Siles et al., 2018; Ai et al., 2019; Dao et al., 2019). For instance, *Phascolarctobacterium* and *Faecalibacterium* are butyrate producers and are considered potentially beneficial bacteria. In this study, these bacteria were decreased in subjects with Crohn’s disease (**Figure 5B**), ulcerative colitis (**Figure 5C**) or irritable bowel syndrome (**Figure 5D**), which would reflect a disruption in intestinal homeostasis (Xu et al., 2021; Zakerska-Banaszak et al., 2021).On the other hand, it is well-known that *Fusobacterium* has been studied widely because of its association with colorectal cancer subjects (Kostic et al., 2012), which is concordant with our results (**Figure 5A**). Interestingly, the study of Guo et al., 2019 suggested an inverse proportion of *Fusobacterium* and *Faecalibacterium* between patients with Crohn’s disease and control subjects; which is consistent with our results for subjects with this disease and their respective controls (**Figure 5B**) (Guo et al., 2019).

The genus *[Ruminococcus] gnavus group* was significantly higher in the group of subjects with Crohn’s disease and irritable bowel syndrome (**Figures 5B, D**). The beneficial role of the Lachnospiraceae family has been debated since some taxa such as *[Ruminococcus] gnavus group* have been reported in high abundance in certain disease states (Vacca et al., 2020). Previous studies have indicated that the association between this genus and Crohn’s disease may be due to the production of a pro-inflammatory polysaccharide by this bacterium (Hall et al., 2017). Nevertheless, there are certain beneficial taxa belonging to this same family that have been identified in the recovery phases of certain diseases that affect intestinal homeostasis, as is the case of *Sellimonas intestinalis* (Muñoz et al., 2020). Finally, the finding of *[Clostridium] innocuum group* as one of the genera enriched in subjects with Crohn’s disease and subjects with ulcerative colitis would allow us to highlight this genus as one of the bacterial communities of importance in inflammatory bowel disease (IBD). Some studies have highlighted *C. innocuum* as an important pathogen, considering that it is resistant to vancomycin and has been reported in patients with Crohn’s disease and ulcerative colitis causing antibiotic-associated diarrhea, severe colitis, and extra-intestinal infections (Chia et al., 2018; Cherny et al., 2021; Le et al., 2022).

As stated above, this study supports previous findings that have been developed in the study of microbiota composition in intestinal inflammatory disease scenarios. Some of the differentially abundant bacteria identified in this study have already been extensively researched for either their beneficial (i.e., *Faecalibacterium*) (Sokol et al., 2008; Ferreira-Halder et al., 2017; He et al., 2021) or potentially pathogenic characteristics (i.e., *Fusobacterium*) (Kostic et al., 2012; Amitay et al., 2017; Zhou et al., 2018b). Hence, our findings confirm the reports of previous studies regarding these bacteria’s relevance in the intestinal microbiota. Alternatively, we also identified some differentially abundant bacteria that have not been as studied as those mentioned above (e.g., *Flavonifractor*, *Subdogranulum*, and *Romboutsia*, among others). Thus, it is necessary to investigate these bacteria that may be relevant in the disease states of the intestinal microbiota with further detail. Moreover, we noticed some bacteria that were differentially abundant within the diseases. For example, the genus *Peptostreptococcus* displayed an increased abundance in colorectal cancer subjects from three of the five colorectal cancer studies, suggesting the relevance of this bacterium in this particular disease (**Figure 5A**). The mentioned result contrasts with recent findings in which subjects with colorectal cancer present enrichment of *Peptostreptococcus* (Osman et al., 2021; Zhang et al., 2021), and it has even been suggested that this bacterium may co-occur with *Fusobacterium* (Kwong et al., 2018).

In addition, it should be considered that some of these differentially abundant bacteria found herein may be present in the subjects due to the imbalance that characterizes the host’s disease. Another option to consider is that these differentially abundant bacteria may be exerting a negative effect on the host’s health, either exacerbating the pre-existing condition or having an association with the disease progression. Apart from this, oxidative stress is often a scenario in intestinal inflammatory diseases such as IBD or colorectal cancer (Dam et al., 2019; Basak et al., 2020), where gut epithelial cells produce reactive oxygen species and reactive nitrogen species during these inflammatory responses (Gibson et al., 2014). Consequently, there is a shift from anaerobic to higher proportions of aerotolerant or facultative anaerobic taxa (Dam et al., 2019). Thus, these facultative anaerobe species may have an advantage compared to the other anaerobic commensals of the healthy gut, as they tolerate the increased oxidative stress. In fact, *Ruminococcus gnavus*, a differentially abundant bacteria found herein, has been reported as a bacterium able to cope with oxidative stress in an IBD scenario (Hall et al., 2017).

Nonetheless, this study has some limitations, such as a low number of studies in some of the considered diseases, due to the availability of sequencing data or the quality of the sequences available for analysis. On the other hand, it has been reported that particular pairs of primers have greater efficiency in amplifying the hypervariable regions of the 16s-rRNA, which in turn would provide a more accurate description of the bacterial communities of the intestinal microbiota (Mancabelli et al., 2017). However, the present study did not consider a unique hypervariable region of 16s-rRNA or a specific pair of primers as query criteria because it tended to reduce the volume of data to be analyzed. The taxonomic classification and the abundance of bacterial taxa may vary according to the targeted hypervariable region of the 16s-rRNA (Tremblay et al., 2015). For instance, when contrasting V3-V4 and V1-V2, the latter may not detect some bacterial taxa adequately, and some taxa may have an overestimated diversity (Seedorf et al., 2014; Chen et al., 2019). Therefore, it has been suggested that targeting V1-V4 provides a higher resolution for bacterial clustering (Chen et al., 2019). In this study, each disease has a common hypervariable region in most studies. For example, the colorectal cancer studies primarily sequenced the V1-V2 region, while the studies of Crohn’s disease, ulcerative colitis, and irritable bowel syndrome concentrated on sequencing the V3-V4 or V4 region (**Supplementary Data 1**). Although the phylogenetic resolution of these regions is different, it provides information in this first approach to identify those genera that are differentially abundant in inflammatory intestinal diseases (Yang et al., 2016). Also, future research in microbiota should unify the criteria for selecting hypervariable regions to describe the bacterial composition of the microbiota.

Prospective studies conducted on clarifying both pathogenic and beneficial mechanisms in host-microbiota interactions should be carried out in large populations, both cross-sectionally and longitudinally, and considering host factors such as environmental factors, metabolome, transcriptome, genotype, etc. Likewise, these studies can use multiple omics analyzes and sophisticated computational tools to correlate the candidates as biomarkers to a disease scenario (Metwaly et al., 2022). For instance, the complete sequencing of the 16s-rRNA should be considered as an approach for the description of bacterial communities as this allows a taxonomic assignment at the species level and with it a better understanding of these communities in the intestinal microbiota (Matsuo et al., 2021). Alternatively, a metagenomic approach for the microbiome characterization would provide a volume of information necessary to describe the composition microbiota communities, obtain information about their functionality, and even assemble the genomes of certain taxa.

Genome assembly has become a convenient approach for studying the intra-taxa diversity of bacterial communities, considering that differential functional profiles may exist in these subpopulations. An example of this is the phylogroups of *Faecalibacterium prausnitzii* present different antibiotic resistance markers and differential modulation of anti- and pro-inflammatory interleukins (Martín et al., 2017). In contrast, genomic analyzes have also been applied to characterize pathogenic variants, such as the case of *Fusobacterium varium* Fv113-g1, a variant that presents paralogs of the FadA adhesin that contribute to its virulence by stimulating the inflammatory and oncogenic response in its host (Sekizuka et al., 2017). As a final step to associate biomarkers to an intestinal pathology, future research should perform functional validation of these biomarkers employing *in vivo* and *in vitro* procedures (Metwaly et al., 2022).

We propose that the 25 bacterial taxa found herein could be denominated as differentially abundant bacteria with relevance in intestinal inflammatory diseases and not as biomarkers. As mentioned before, biomarkers are understood as those taxa that may be related to a type of diet, lifestyles of the host, or disease states. However, it was also proposed that the term biomarkers apply to the description of those dominant taxa in a community (Gorvitovskaia et al., 2016). Therefore, this term can lead to confusion because the most abundant taxa are not necessarily those that play a crucial role in states of health or disease of the host. Additionally, the concept of biomarkers would be affected by the intra-taxa diversity of important bacterial communities, considering that within a species, there may be functional characteristics that would affect the intestinal microbiota and microbiome differentially (Segata, 2018).

Given the above, it is necessary to redefine or expand the current definition of biomarkers, where those bacterial communities with a differential presence or abundance in a given scenario are considered, as well as the evaluation of the functional differences that may occur at the intra-taxa level. Also, considering that a clinical biomarker should be quantitative, reproducible, and exhibit accuracy in predicting a disease across large populations or ethnicities (Metwaly et al., 2022). However, when associating particular bacterial taxa to some disease state of the host, the following variables should be considered: evaluation of genetic, metabolic, and immunological factors of the host, as well as the interactions that these taxa could have with other members of the microbiota. In addition to this, it is essential to define a consensus statistical method to identify these bacterial communities differentially present in some specific health scenarios. Evidence of the previously stated is the low concordance in the differentially abundant genera contrasted between the original study and the results obtained here, especially in the case of ulcerative colitis and Crohn’s disease (**Supplementary Data 2**). The variation of the results may be in function of the methodology used, from the refinement of the sequences to the taxonomic assignment, as well as the approach used to identify those differentially abundant bacteria, with LEfSe being one of the most employed by studies originals.

The identification of these relevant bacterial communities in the intestinal microbiota would allow expanding our knowledge about the association of certain health and disease states and the composition of the intestinal microbiota. The candidates of important bacterial communities could be proposed as next-generation probiotics (NPGs), considering that NGPs are microorganisms that had not previously been used as agents to promote health states, and even those genetically modified microorganisms are included in this group. With new sequencing and genetic modification technologies, the identification of these important bacterial communities could contribute to the development of NGPs, especially in the design of therapies with NPGs aimed at treating specific diseases (O’Toole et al., 2017; Chang et al., 2019).

## Data availability statement

The datasets presented in this study can be found in online repositories. The names of the repository/repositories and accession number(s) can be found in the article/**Supplementary Material**.

## Author contributions

MM, JDR, and LV defined the approach to perform the query in the databases. LV and LB performed the data processing, analyses, and data visualization. MM and JDR revised and edited the manuscript. All authors contributed to the article and approved the submitted version.

## Acknowledgments

We acknowledge Giovanny Herrera for supporting the approach of this research.

## Conflict of interest

The authors declare that the research was conducted in the absence of any commercial or financial relationships that could be construed as a potential conflict of interest.

## Publisher’s note

All claims expressed in this article are solely those of the authors and do not necessarily represent those of their affiliated organizations, or those of the publisher, the editors and the reviewers. Any product that may be evaluated in this article, or claim that may be made by its manufacturer, is not guaranteed or endorsed by the publisher.
