# Supplementary material for: Do we need to change our perspective about gut biomarkers? A public data mining approach to identify differentially abundant bacteria in intestinal inflammatory diseases
Source: Front Cell Infect Microbiol. 2022 Nov 21;12:918237. doi: 10.3389/fcimb.2022.918237 (PMC9719923; doi:10.3389/fcimb.2022.918237)
Supplement: Supplementary file 1 [file DataSheet_1.pdf]

Supplementary Material

Do we need to change our perspective about gut biomarkers? A public data mining approach to identify differentially abundant bacteria in intestinal inflammatory diseases

Laura Vega[1], Laura Bohórquez[1], Juan David Ramírez [1,2], Marina Muñoz[1,*]

[1] Centro de Investigaciones en Microbiología y Biotecnología-UR (CIMBIUR), Facultad de Ciencias Naturales, Universidad del Rosario, Bogotá, Colombia

[2] Molecular Microbiology Laboratory, Department of Pathology, Molecular and Cell-Based Medicine, Icahn School of Medicine at Mount Sinai, New York, NY 10029, USA.

*** Correspondence:**
Corresponding Author
claudia.muñoz@urosario.edu.co

1 Supplementary Data

Supplementary Data 1. Dataset of the studies selected by disease, including fields such as the author, study title, year of publication, disease on which the study is focused, name of the repository, study accession number, type of samples taken in the study, sequencing platform, hypervariable regions of the 16s-rRNA sequenced, set of primers implemented for sequencing, the number of samples considered, and additional observations.

Supplementary Data 2. Comparison of the bacteria identified as differentially abundant in the original study and in the results of this study. The table mentions the approach used by the original study to detect differentially abundant bacteria between the study groups. Additionally, the table includes a field of the shared bacteria between the original study and the results herein.

2 Supplementary Figures

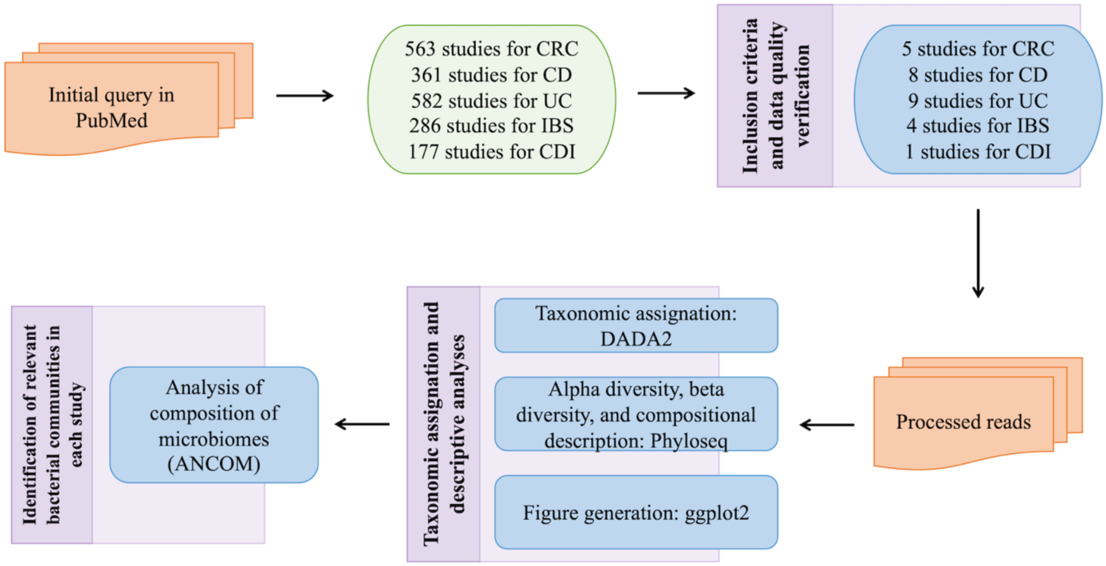

Supplementary Figure 1. Summary of the processes implemented here to analyze public data of microbiota studies. Initially, we performed a query in PubMed for each of the intestinal inflammatory diseases. After the inclusion criteria and quality verification we retrieved a total of 27 studies. We performed the removal of barcodes from the sequences if necessary. Afterwards, we implemented DADA2 package for the taxonomic assignation and phyloseq package for microbiota analyses (alpha and beta diversity). Finally, we performed an Analysis of composition of microbiomes (ANCOM) to identify differentially abundant bacteria between the two study groups (cases and controls) of the considered diseases.
